# Mirror self-recognition in gorillas (*Gorilla gorilla gorilla*): a review and evaluation of mark test replications and variants

**DOI:** 10.1007/s10071-021-01592-3

**Published:** 2022-01-07

**Authors:** Lindsay E. Murray, James R. Anderson, Gordon G. Gallup

**Affiliations:** 1 School of Psychology, University of Chester, Parkgate Road, Chester, CH1 4BJ UK; 2 Department of Psychology, Kyoto University Graduate School of Letters, Kyoto, Japan; 3 Department of Psychology, University at Albany, State University of New York, Albany, USA

**Keywords:** Evaluation, Gorilla, Method, Mirror self-recognition, MSR, Replication

## Abstract

Mirror self-recognition (MSR), widely regarded as an indicator of self-awareness, has not been demonstrated consistently in gorillas. We aimed to examine this issue by setting out a method to evaluate gorilla self-recognition studies that is objective, quantifiable, and easy to replicate. Using Suarez and Gallup’s (J Hum Evol 10:175–183, 1981) study as a reference point, we drew up a list of 15 methodological criteria and assigned scores to all published studies of gorilla MSR for both methodology and outcomes. Key features of studies finding both mark-directed and spontaneous self-directed responses included visually inaccessible marks, controls for tactile and olfactory cues, subjects who were at least 5 years old, and clearly distinguishing between responses in front of versus away from the mirror. Additional important criteria include videotaping the tests, having more than one subject, subjects with adequate social rearing, reporting post-marking observations with mirror absent, and giving mirror exposure in a social versus individual setting. Our prediction that MSR studies would obtain progressively higher scores as procedures and behavioural coding practices improved over time was supported for methods, but not for outcomes. These findings illustrate that methodological rigour does not guarantee stronger evidence of self-recognition in gorillas; methodological differences alone do not explain the inconsistent evidence for MSR in gorillas. By implication, it might be suggested that, in general, gorillas do not show compelling evidence of MSR. We advocate that future MSR studies incorporate the same criteria to optimize the quality of attempts to clarify the self-recognition abilities of gorillas as well as other species.

## Introduction

Mirror self-recognition (MSR), widely regarded as an indicator of self-awareness, has been studied in many species, notably primates, with mixed results. Although the strongest evidence for MSR in nonhumans has been found in great apes, one of the most perplexing species is the western lowland gorilla (*Gorilla gorilla gorilla*); positive evidence for MSR in gorillas is less consistently reported than for chimpanzees and orangutans. Our aim in this paper was to examine this issue by proposing a method to evaluate gorilla self-recognition studies that is objective, quantifiable, and easy to replicate. This method can be used as a form of quality control for MSR studies in other species too.

Although earlier, largely anecdotal descriptions of gorillas’ reactions to their reflections existed (Benchley 1944; Hoyt 1941; Riopelle 1970; Yerkes 1927), Lethmate (1974) was the first to replicate the effects of the mark test, a systematic procedure introduced in Gallup’s (1970) study of MSR in chimpanzees and three species of macaques. In the original mark test, the subject was anesthetized and then marked on a normally unseen body part (e.g. head, ear), observed in the absence of the mirror to record any spontaneous responses to the mark, and finally observed again in front of the mirror. Individuals who pass the mark test typically touch the mark while looking in the mirror or just afterwards; they often then look at and may smell their fingers. Suarez and Gallup (1981) introduced improvements to Gallup’s (1970) original mirror exposure and mark test procedure by including a condition where anesthetized gorillas were not only marked on their faces but also their wrists, thus providing a control condition that can be used to demonstrate that the gorillas would in fact be interested in comparable marks on their faces if they were capable of mirror self-recognition. This is, therefore, the study we use as the reference point for evaluating all studies of gorilla self-recognition.

The literature on primate MSR since Suarez and Gallup (1981) is characterized by huge variability in target species, settings, methods, procedures, quality of evidence, and interpretations of the data. This variability has contributed to the mixed picture regarding MSR in gorillas. In addition, new theoretical perspectives have emerged; for example, relating MSR to neuroanatomical mechanisms (Hecht et al. 2017), which complement older approaches such as the “clambering hypothesis,” which proposed the evolution of an awareness of personal agency for safe arboreal locomotion in large-bodied primates (Povinelli and Cant 1995). Gallup (1997) elaborated on this hypothesis, suggesting that whereas self-awareness was preserved in orangutans because of their arboreality, evolutionary developments were different for other ape species which became more terrestrial. For example, humans used self-awareness to compete among one another for scarce resources, gorillas may have lost the capacity due to genetic drift, while chimpanzees may even be in the process of losing the capacity. These hypotheses address both inter- and intra-species differences in MSR. Given that gorillas’ sensorimotor and locomotor developmental trajectories differ from those of other great apes (Watts and Pusey 1993), it has been argued that gorillas’ increasing terrestriality may have resulted in the evolving ancestral capacity for self-recognition being “turned off” (Povinelli 1993). An alternative suggestion is that the capacity for MSR evolved gradually, in incremental steps, rather than as an either/or emergence (de Waal 2019; Murray 2020; Murray et al. 2020). For example, gorillas’ responses to their live video images have been categorized according to a continuum of different levels of self-recognition (Murray 2020).

In the absence of any systematic analysis of studies of gorillas’ responses to reflections, we examined the literature on gorilla MSR capacities in more detail. To this aim, we developed an evaluation procedure in which scores were assigned for key methodological features and for the outcomes of the studies, yielding an overall score for each study. We predicted, first, that MSR studies would score progressively higher as procedures, including behavioural coding methods, improve over time. Second, if improved procedures increase the likelihood of demonstrating MSR in gorillas, then the correlation between scores for procedures and outcomes should likewise be positive.

## Methods

With reference to Suarez and Gallup (1981), we compiled a list of 15 methodological criteria and then scored all published studies of MSR in gorillas according to this list (see Table 1 for criteria and the rationale for each). Note that we included the early anecdotes of Yerkes (1927), Benchley (1944), Hoyt (1941), and Riopelle (1970) due to the scarcity of systematic studies. Each study received 1 point for each criterion that was met, 0 for each that was not met, or no score if details were not sufficiently clear.

**Table 1:** Methodological criteria used to evaluate gorilla MSR studies

Criterion	Description	Rationale
1	Group (defined as ≥ 2) vs. individual mirror exposure	Provides more information about source of reflections (Gallup and Anderson 2018)
2	Minimum of 50 h’ mirror exposure	Provides enough time for subject to learn to self-recognize
3	Use of angled mirrors or televised live images instead of mirror	Reduces eye contact and hence gaze aversion (Anderson and Roeder 1989; Shillito et al. 1999)
4	More than one subject tested	Differentiates those studies where several individuals have mirror access but only some are tested; accounts for individual variation
5	Sessions videotaped and available for inspection	Provides lasting evidence for scrutiny
6	Videos coded by ‘blind’ raters	Provides independence of interpretation of responses
7	Subject(s) adequately socially reared, displaying relative normal behaviours	Chimpanzees reared in isolation fail to self-recognize (Gallup et al. 1971)
8	Subject(s) mature enough (defined as ≥ 5 years) for MSR	Eliminates individuals not expected to achieve MSR due to immaturity
	*Additional criteria for mark test studies*	
9	Use of general anaesthetic or sham marking	Prevents knowledge about presence of the marks without a mirror
10	Non-directly visible marks (e.g. on head) applied	Tests the use of mirror information about the self
11	Control for tactile and olfactory cues	Prevents contamination from extraneous cues
12	Directly visible control marks applied	Tests motivation to touch marks
13	Post-marking observations, no mirror	Tests spontaneous responses, without a mirror, as baseline control for responses when mirror present
14	Absence of humans during the test	Prevents contamination of results due to facilitation, distraction, or behavioural inhibition (Patterson and Cohn 1994)
15	Distinguishing between mark-directed responses in front of vs. away from mirror	Tests spontaneous responses to mark, without a mirror, as control for responses to mark when mirror present

Following initial piloting of these 15 methodological criteria, we added more weight to evidence of mirror-mediated self-directed and mark-directed behaviour. For example, the Shillito et al. (1999) gorilla study received 10 points—more than Gallup’s (1970) study of chimpanzees—despite finding no evidence of MSR in gorillas. Therefore, we awarded 5 points for spontaneous, mirror-guided, self-directed responses (i.e. using the reflection to investigate body parts that otherwise cannot be seen), and 10 points for unambiguous mark-directed responses while looking in the mirror.

For studies that included no mark test, only the first eight criteria were relevant, and with up to 5 points for evidence of mirror-guided self-directed responses, these studies could obtain a maximum of 13 points. For studies incorporating mark tests, all 15 criteria were relevant and, with up to 5 points for spontaneous self-directed responses and 10 points for positive mark-directed responses, the maximum possible score was 30 points. Applying this scoring system thus gave every published gorilla MSR study a methods score and an outcome score, in addition to the total score, which might be regarded as a “quality” score. For ease of comparisons between studies (those using the mark test and those not), scores were also converted to percentages of possible maximum score.

We used Spearman’s rank order correlation to test our prediction that MSR methods would improve over time. Correlations were also used to test whether studies with higher methods scores yielded higher outcome scores, whether more positive findings were published more recently, and whether the overall percentage scores improved over time.

## Results

Table 2 shows the 21 gorilla MSR studies included in the evaluation, with a brief description of their methods and the main outcomes. Fifteen studies employed a mark test and involved one to six subjects including male and female adults (note that Shillito et al.’s (1999) four experiments are treated as separate studies). Six studies described responses to mirrors (or equivalents) but conducted no mark test, and these involved one to four subjects, including males and females and some younger individuals. Typical responses to reflections included interest, social responses (sometimes decreasing over time) and self-directed behaviours such as mirror-mediated examination of body parts. Mark tests gave rise either to touching or not touching the target mark.

**Table 2:** Descriptions of gorilla MSR studies

Study	*N*	Summary of methods	Findings
*Studies employing the Mark test*			
Lethmate (1974)	6	Extended exposure to mirror for 6 gorillas; 4 individuals mark-tested	Mirror: 2 gorillas used mirror while picking teeth or manipulating other body part. Mark test: 2 of 4 gorillas tested exhibited self-recognition
Suarez and Gallup (1981)	4	1 male (19 years) and 3 females (13, 17 and 18 years); 16 days × 5 h mirror exposure; marked under anaesthetic; 30-min baseline no mirror and 30-min mirror observations after marking	Mirror: viewing and social responses decreased; no self-directed behaviours. Mark test: no mark touching, despite showing interest in control marks on wrists
Ledbetter and Basen (1982)	2	1 male (10 years) and 1 female (11 years); 400 h of exposure to mirrors; marked under anaesthetic; 15-min baseline no mirror and 15-min mirror observations after marking	Mirror: social responses decreased; no self-directed behaviours. Mark test: no mark touching
Parker (1994)	6	Adult male and female in group of six; 17–41-min sessions of mirror exposure	Mark test: 1978: Female Pogo (inadvertently marked by self) and male Bwana (marked by author) both wiped off marks while looking in mirror, the latter with a tool; 1989: self-directed behaviour
Patterson (1978); Patterson and Cohn (1994)	2	Anecdotal accounts of Koko and Michael when exposed regularly to mirrors; mark test without anaesthesia	Mirror: mirror-guided self-directed behaviours from 3.5 y; self-grooming and putting make-up and accessories on in front of a mirror; photographing her mirror image. Mark test: self-directed and mark-directed responses
Evans (cited in Swartz and Evans, 1994)	1	Single male (King, 22 years); marked by keeper	Mark test: self-directed behaviour (but no baseline); touched mark and smelled fingers^a^
Swartz and Evans (1994)	2	1 male (Etoumbi, 14 years) given 80 h of mirror exposure and 1 female (Zoe, 5 years) given 12 h of mirror exposure; 1-h mark test	Mirror: decreasing interest; Mark test: self-directed behaviour, no mark touching
Nicholson and Gould (1995)	1	Single female (Muke, 26 years) trained to find stimulus only visible in mirror	Mirror: interest; Mark test: self-directed and mark-directed behaviour
Shillito et al. (1999)	2	Subjects: 1 male (Mopie, 22 years) and 1 female (Mandara, 12 years, with 4 years of prior mirror experience) Expt 1: given 15–17.5 h of angled mirror exposure; sham-marking for mark test	Expt 1 Mirror: little mirror interest, no body exploration using mirror; Mark test: no self-directed or mark-directed behaviour
Shillito et al. (1999)	2	Subjects: 1 male (Mopie, 22 years) and 1 female (Mandara, 12 years, with 4 years of prior mirror experience) Expt 2: given additional approx. 9 h of normal mirror exposure; sham-marking for mark test	Expt 2 Mirror: some mirror interest; Mark test: Mandara touched marked brow but not while looking in mirror
Shillito et al. (1999)	2	Subjects: 1 male (Mopie, 22 years) and 1 female (Mandara, 12 y, with 4 years of prior mirror experience) Expt 3: given additional approx. 4 h of normal mirror exposure; sham-marking for mark test, no human presence, recorded by video	Expt 3 Mark test: both gorillas touched marked brow but not notably more so in presence of mirror
Shillito et al. (1999)	2	Subjects: 1 male (Mopie, 22 years) and 1 female (Mandara, 12 years, with 4 years of prior mirror experience) Expt 4: marked on wrist as control	Expt 4 No mirror; Mark test: both gorillas showed interest in wrist marks
Shumaker and Swartz (2002)	1	Single male (Mopie, 25 years); trained to remove dots from enclosure and self, then to touch a laser dot	Mark test: used mirror to guide hand to remove dot sticker and to touch laser spot
Posada and Colell (2007)	1	Single male (Xebo, 17 years); 28 h exposure to mirror; marked by keeper; 30-min no mirror baseline after marking and 45-min mark test observation with mirror	Mirror: interest; no agonistic behaviour; ‘self-referred’ action (including new body postures) and ‘pulling face’ responses. Mark test: no baseline self-directed action; touched mark first away from mirror, smelled fingers, then touched mark in front of mirror and smelled fingers; immediately wiped away control marks
Allen and Schwartz (2008)	1	Single male (Otto, 45 years); 22.5 h exposure to mirror; marked by keeper; 5 × 30-min sham mark trials; 3 × 30-min paint test trials	Mirror: no clear details. Mark test: found mark ‘accidentally’ when touched a water bottle to face and transferred paint to bottle; mirror-mark-directed behaviours in test trials but not sham trials; some mirror-guided behaviour
*Studies not employing the Mark test*			
Yerkes (1927)	1	Anecdotal description of response of female mountain gorilla, Congo (5 years), to mirror	Congo described as showing interest, touching glass, looking and feeling behind mirror
Hoyt (1941)	1	Anecdotal description of response of home-reared gorilla, Toto, to mirror	Toto described as preening herself and examining teeth, but also attacking mirror
Benchley (1944)	2	Anecdotal description of response of mature zoo-living mountain gorillas (Mbongo and Ngagi) to reflection in pool	Gorilla described as displaying and splashing water
Riopelle (1970)	2	Anecdotal account of albino gorilla, Snowflake, and another gorilla, Muni, both aged 6 years, when exposed briefly to mirrors	Mirror: Snowflake fled and then beat on his reflection with bared teeth (social response); Muni “…examined parts of his body that he cannot ordinarily see.” (p. 500) = self-directed behaviour
Law and Lock (1994); Murray (2020)	4	2 males (5 and 26 years) and 2 females (17 and 26 years) each presented with approx. 30 min of video stimuli, including presentation of recorded and live self-images	Video: self-directed behaviour, notably from one juvenile male (included looking inside mouth)
Inoue-Nakamura (1997)	1	Single female (12 years); 25-min exposure to mirror	Mirror: self-directed behaviours

^a^One of the authors (GG) has seen the video upon which this claim is made, and is unconvinced that it shows evidence of mirror-mediated mark-directed behaviour

Table 3 shows the scores for methods, outcomes and overall total for each study. For studies including a mark test, total percentage scores (where 100% would be the highest scoring, therefore, highest quality, study) ranged from 20 to 80%, with a mean of 47%. Therefore, with Allen and Schwartz (2008) receiving the highest score of 80%, and Shillito et al.’s (1999) Experiment 4 receiving the lowest score of 20%, 9 of the 15 studies scored on or above average, and 6 scored below. For studies with no mark test, total percentage scores ranged from 8 to 85% (mean: 48.8%), with Yerkes (1927) and Benchley (1944) scoring below average and the remaining four studies scoring above average. Methods scores for studies using the mark test ranged from 2 to 10 out of 15, with a mean of 7.10. For studies with no mark test methods, scores ranged from 1 to 6 out of 8, with a mean of 3. In studies using the mark test, 8/15 (53%) reported self-directed behaviour and 8/15 (53%) reported mark-directed behaviour. All studies that reported mark-directed responses also reported self-directed responses, with two exceptions: Swartz and Evans (1994) reported only self-directed responses, and Shumaker and Swartz (2002) reported mark-directed responses without self-directed behaviour. In studies not including a mark test, 4/6 (67%) reported self-directed behaviour.

**Table 3:** Quantitative evaluation of gorilla MSR studies

Study	Findings		Methodological criteria															Methods total	Overall total	Overall %
			1	2	3	4	5	6	7	8	9	10	11	12	13	14	15			
*Studies employing the Mark test*	Self-directed responses^a^ (+5 points)	Mark test responses (+10 points)																		
Lethmate (1974)	5	10	1	–	0	1	0	0	–	–	–	–	–	–	–	–	–	2	17	57
Suarez and Gallup (1981)	0	0	1	1	0	1	0	0	–	1	1	1	1	1	1	0	1	10	10	33
Ledbetter and Basen (1982)	0	0	1	1	0	1	0	0	–	1	1	1	0	0	1	0	0	7	7	23
Parker (1994)	5	10	1	0	0	1	1	0	1	1	0	1	0	0	0	0	0	6	21	70
Patterson (1978); Patterson and Cohn (1994)^b^	5	10	0	1	0	0	0	1	0	1	1	1	1	0	0	0	1	7	22	73
Evans (cited in Swartz and Evans 1994)^b^	5	10	0	0	0	0	0	0	0	1	1	1	1	0	1	0	1	6	21	70
Swartz and Evans (1994)	5	0	0	1	0	1	0	0	1	1	1	1	1	1	1	0	0	9	14	47
Nicholson and Gould (1995)	5	10—but not clear	0	0	0	0	0	0	0	1	0	1	1	0	1	0	1	5	20	67
Shillito et al.—Expt 1 (1999)	0	0	1	0	1	1	0	0	0	1	1	1	1	0	1	0	1	9	9	30
Shillito et al.—Expt 2 (1999)	0	0	1	0	0	1	0	0	0	1	1	1	1	0	1	0	1	8	8	27
Shillito et al.—Expt 3 (1999)	0	0	1	0	0	1	1	–	0	1	1	1	1	0	1	1	1	10	10	33
Shillito et al.—Expt 4 (1999)	0	0	1	0	0	1	0	0	0	1	0	0	1	1	1	0	0	6	6	20
Shumaker and Swartz (2002)	0	10	0	0	0	0	0	0	0	1	1	1	1	0	0	0	0	4	14	47
Posada and Colell (2007)	5	10	0	0	0	0	1	0	1	1	0	1	1	1	1	0	1	8	23	77
Allen and Schwartz (2008)	5	10	0	0	0	0	1	1	0	1	1	1	1	0	1	1	1	9	24	80
*Studies not employing the Mark test*	Self-directed responses (+5 points)																			
Yerkes (1927)	0		0	0	0	0	0	0	–	1	0	0	0	0	0	0	0	1	1	8
Hoyt (1941)	5		0	1	0	0	0	0	0	1	0	0	0	0	0	0	0	2	7	54
Benchley (1944)	0		1	0	0	0	0	0	0	1	0	0	0	0	0	0	0	2	2	15
Riopelle (1970)	5		1	0	0	1	0	0	1	1	0	0	0	0	0	0	0	4	9	69
Law and Lock (1994) /Murray (2020)	5		1	0	1	1	1	0	1	1	0	0	0	0	0	0	0	6	11	85
Inoue-Nakamura (1997)	5		1	0	0	0	1	0	-	1	0	0	0	0	0	0	0	3	8	62

^a^Self-directed behavioural responses to mirrors outside of the mark test context

^b^Both Patterson and Swartz & Evans have refused to comply with reasonable requests from GG to examine their videotapes, so have not been assigned a score for this criterion

To highlight the criteria most associated with successful demonstrations of gorilla MSR, we calculated the number of studies using each of the 15 criteria. Table 4 shows that mark-directed responses were seen particularly in studies involving visually inaccessible marks, controls for tactile and olfactory cues, subjects who were at least five years old, and a clear distinction between mark-directed responses in front of versus away from the mirror. These criteria are also key features of studies finding self-directed responses. Additional criteria that appear important for the quality of studies include videotaping the tests, having more than one subject, testing subjects with adequate social rearing, reporting post-marking observations with mirror absent, and giving mirror exposure in a social versus individual setting.

**Table 4:** Most frequently included criteria in studies reporting self-directed and mark-directed responses to mirrors in gorillas (%)

Criteria	Mark-directed responses	Self-directed responses
Hidden Marks	29.17	12.96
Mature subjects	25.00	20.37
Cue control	25.00	11.11
Front v away from mirror	20.83	9.26
Group mirror exposure		9.26
More than one subject		9.26
Video recordings of responses		9.26
Social grouping		9.26
Post-mark observations		9.26

**Fig. 1:**
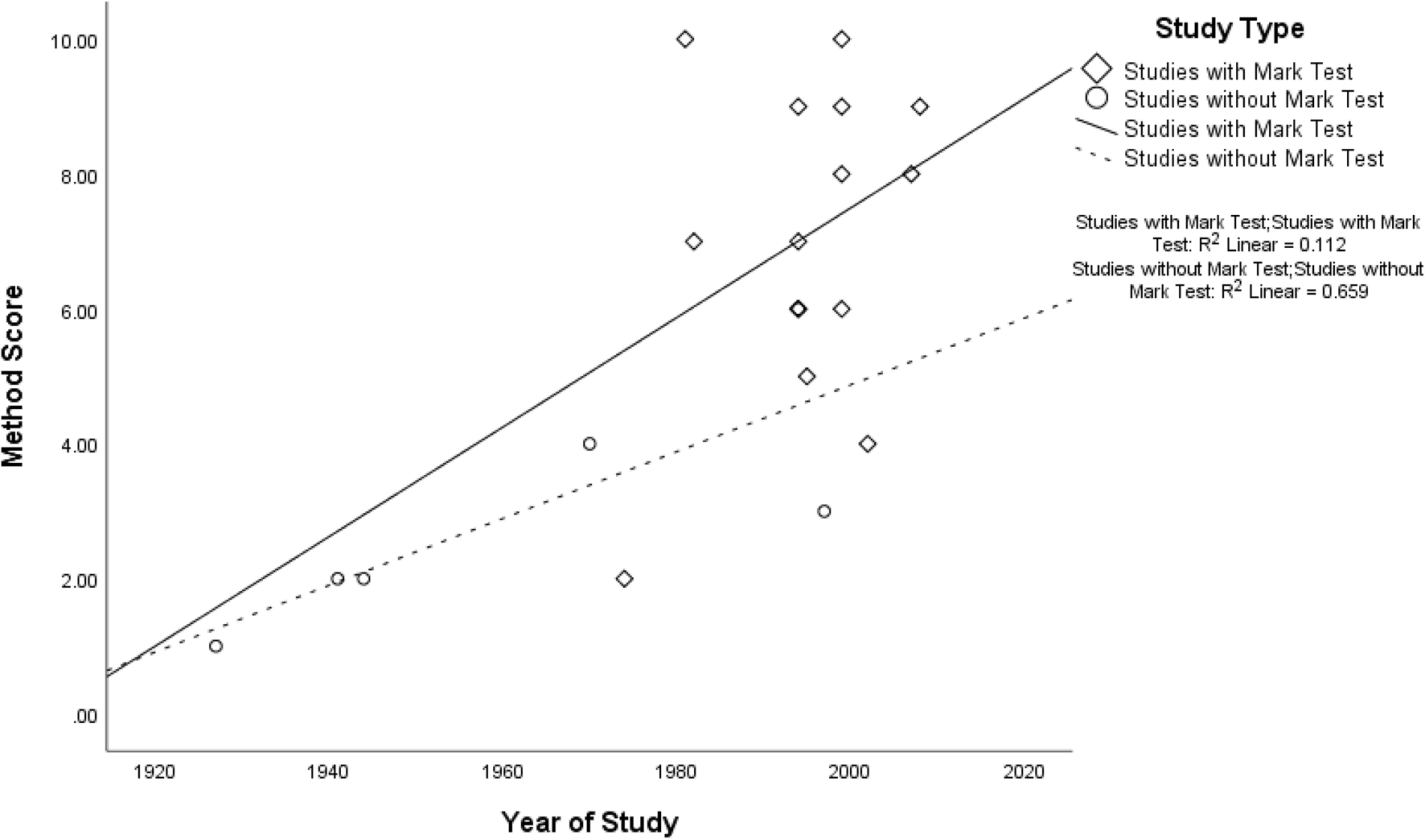
Correlation between year of MSR study and methods score

The Spearman’s correlation coefficient was positive and significant between methods score and year of study (*r*_s_ = 0.55, *N* = 21, *p* = 0.005), demonstrating that, over time, studies became more methodologically rigorous. Figure 1 shows that this is the case whether MSR studies included the mark test or not. However, higher scores on methodological rigour did not correlate with higher scores for outcome (*r*_s_ = − 0.13, *N* = 21, *p* = 0.29), and the latter were not related to year of publication of the study (*r*_s_ = 0.20, *N* = 21, *p* = 0.20). Finally, there was no significant correlation between overall total scores and year of study (*r*_s_ = 0.27, *N* = 21, *p* = 0.12).

## Discussion

We found a wide range of scores in our assessment of the quality of MSR studies in gorillas. Most of the studies with no mark test reported self-directed behaviour, while just over half of the mark test studies reported both self-directed and mark-directed behaviour. Over time, studies—with or without the mark test—have become methodologically more rigorous; however, this has not led to more positive outcomes. We found no link between when studies were conducted and either outcome or total scores (methodology and findings). Our prediction that studies would obtain progressively higher total scores as procedures and behavioural coding methods improved was not supported. However, when looking at the methodological criteria alone, the prediction was supported, as scores for methodological rigour did increase over time. While methodological rigour is clearly important, improvements in methods do not guarantee stronger evidence of self-recognition in gorillas. This lack of association could be taken as evidence that, at the species level, gorillas do not show compelling evidence of MSR. Alternatively, it may reflect wide intra-species variability. Like many studies on various aspects of cognition, most gorilla MSR studies have small sample sizes. Much remains unknown about how other factors, such as rearing, experience and setting, interact with basic individual differences in self-recognition propensity.

Awarding additional points for positive instances of both self-directed and mark-directed responses revealed that studies with no such responses received a low score, even if the method score was high, a trend reflected in the negative but non-significant correlation between methods score and outcome. Looking only at the methods totals (the 15 criteria), it is clear that the reference study (Suarez and Gallup 1981) scores the highest (10 out of 15 points), along with Shillito et al.’s (1999) Experiment 3. As methodologically stronger studies do not appear to yield more evidence of self-recognition in gorillas, procedural details seem unlikely to explain why positive evidence is so modest (de Veer and van den Bos 1999), although some authors have criticized use of a ‘chimpanzee standard’ to investigate MSR across species (Shumaker and Swartz 2002). Here, the argument is that the frequent failure of gorillas to pass the mark test may be due to as yet unidentified limitations of the mark test for revealing self-recognition in this species.

Contrary to the criticism of using a chimpanzee standard to investigate MSR in gorillas, it is important to examine those factors associated with positive responses in gorillas. Mark-directed responses occurred in studies involving visually inaccessible marks, tactile and olfactory controls, subjects of at least 5 years of age, and a clear distinction between responses in front of versus away from the mirror. These are clearly important factors which future studies on mirror self-recognition in gorillas should seek to replicate. Although gorillas often fail to respond to marks on their faces that can only be seen in a mirror, they do show an avid interest in comparable control marks on their wrists (Suarez and Gallup 1981). The results of studies that use dyes, stickers, or lasers, as in the trained monkey studies (Chang et al. 2017), have reduced validity as long as there are possible olfactory, tactile, or irritant cues from the marks. Shumaker and Swartz (2002) claimed to have found evidence of MSR in an individual gorilla who had previously failed (Shillito et al. 1999) using a training paradigm involving the use of stickers and lasers. According to these authors, their training procedures provided the necessary motivation for the gorilla to reveal his true ability. But it is important to bear in mind that trained positive outcomes are not the same as spontaneous ones (Gallup and Suarez 1986). Some other MSR studies with gorillas have included specific experimental manipulations designed to facilitate successful self-recognition, including the use of angled mirrors, but without success (Shillito et al. 1999).

Additional important quality-related features of studies reporting mirror-guided self-directed responses include video-recorded tests, more than one subject, subjects with adequate social rearing, post-marking observations with mirror absent, and mirror exposure in a social versus individual setting. It is noteworthy that three gorillas reported to pass the mark test (Patterson and Cohn 1994; Swartz and Evans 1994) were raised in enculturated, enriched environments with extensive human contact, possibly resulting in a latent capacity for self-recognition being “switched back on” (Povinelli 1993). However, these results must be viewed as tenuous because of the lack of public availability of the relevant video evidence.

Gorilla MSR studies often involve removing subjects from their group for mirror exposure (e.g. Swartz and Evans 1994). This separation may negatively affect both those left behind in the group and the separated individual, particularly if they are immature. The emotional response to the separation, coupled with lack of experience in cognitive studies, may lead to attentional and emotional barriers to optimal performance in the test. Allen and Schwartz (2008) suggested that, as their single gorilla ‘passed the test’ without showing prior mirror-guided or contingent behaviours, these may not be pre-requisites. But contingency testing is open to alternative interpretations; for example, the subject may simply be trying to get the other individual in the mirror to reciprocate and respond normally instead of only mimicking the behaviour of the subject (Gallup and Anderson 2020). However, in Allen and Schwartz’s (2008) report, the timings of multiple sham and test trials, and whether the mirror was present or not are often unclear, and so assigning scores was not always easy. To facilitate future evaluations, we recommend that due attention be paid to details when describing methods and observations. These details should include observing and reporting responses in front of versus away from the mirror, and post-marking observations with mirror absent.

It is also important to acknowledge that applicability of our evaluation criteria has changed over time. For example, fewer early studies included video recordings. But with the modern widespread availability of video, hopefully more researchers will be open to sharing footage in response to reasonable requests. Finally, studies should include not just ratings by “blind” observers, but also reports of inter-rater reliability.

In conclusion, we tried to scrutinize every published paper addressing the question of mirror self-recognition in gorillas, examining methodological details both alone and in combination with reported occurrences of self-directed and mark-directed responses. We hope that researchers might heed the criteria used here, particularly those highlighted in Table 4, to optimize the quality of future studies of the self-recognition abilities of gorillas as well as other species.

## Data Availability

Available.
